# Effect of weight loss on cardiometabolic risk: observational analysis of two randomised controlled trials of community weight-loss programmes

**DOI:** 10.3399/bjgp20X714113

**Published:** 2021-03-09

**Authors:** Elizabeth Morris, Susan A Jebb, Jason Oke, Alecia Nickless, Amy Ahern, Emma Boyland, Ian D Caterson, Jason Halford, Hans Hauner, Paul Aveyard

**Affiliations:** Nuffield Department of Primary Care Health Sciences, Radcliffe Observatory Quarter, Woodstock Road; NIHR Oxford Biomedical Research Centre, Oxford, UK.; Nuffield Department of Primary Care Health Sciences, Radcliffe Observatory Quarter, Woodstock Road; NIHR Oxford Biomedical Research Centre, Oxford, UK.; Nuffield Department of Primary Care Health Sciences, Radcliffe Observatory Quarter, Woodstock Road; NIHR Oxford Biomedical Research Centre, Oxford, UK.; Nuffield Department of Primary Care Health Sciences, Radcliffe Observatory Quarter, Woodstock Road, Oxford; Exploristics Ltd., Belfast, UK.; MRC Epidemiology Unit, University of Cambridge School of Clinical Medicine, Cambridge, UK.; Department of Psychology, Institute of Population Health, University of Liverpool, Liverpool, UK.; SoLES, University of Sydney, Boden Collaboration, Charles Perkins Centre, University of Sydney, Australia.; Psychology, University of Leeds, Leeds, UK; president of the European Association for the Study of Obesity.; Chair of nutritional medicine and director of the Else Kröner-Fresenius-Centre of Nutritional Medicine, Technical University of Munich, Munich, Germany.; Nuffield Department of Primary Care Health Sciences, Radcliffe Observatory Quarter, Woodstock Road; NIHR Oxford Biomedical Research Centre, Oxford, UK.

**Keywords:** cardiometabolic risk, diabetes, hypertension, lifestyle, obesity, overweight, primary care, weight loss

## Abstract

**Background:**

Guidelines recommend that clinicians identify individuals at high cardiometabolic risk and support weight loss in those with overweight or obesity. However, we lack individual level data quantifying the benefits of weight change for individuals to guide consultations in primary care.

**Aim:**

To examine how weight change affects cardiometabolic risk factors, and to facilitate shared decision making between patients and clinicians regarding weight loss.

**Design and setting:**

Observational analysis using data from two trials of referral of individuals with overweight or obesity in primary care to community weight-loss groups.

**Method:**

Linear mixed effects regression modelling examining the association between weight change and change in systolic blood pressure (SBP), diastolic blood pressure (DBP), fasting glucose, glycated haemoglobin (HbA1c), and lipid profile across multiple timepoints (baseline to 24 months). Subgroup analyses examined changes in individuals with hypertension, diabetes, and hyperlipidaemia.

**Results:**

In total, 2041 participants had a mean (standard deviation) age of 50 (SD 13.5) years, mean baseline weight of 90.6 (14.8) kg and mean body mass index (BMI) of 32.7 (SD 4.1) kg/m^2^. Mean (SD) weight change was −4.3 (SD 6.0) kg. All outcome measures showed statistically significant improvements. Each 1 kg weight loss was associated with 0.4 mmHg reduction in SBP and 0.3 mmHg reduction in DBP, or 0.5 mmHg and 0.4 mmHg/kg respectively in people with hypertension. Each 1 kg weight loss was associated with 0.2 mmol/mol reduction in HbA1c, or 0.6 mmol/mol in people with diabetes. Effects on plasma lipids were negligible.

**Conclusion:**

Weight loss achieved through referral to community weight-loss programmes, which are commonly accessible in primary care, can lead to clinically relevant reductions in BP and glucose regulation, especially in those at highest risk.

## INTRODUCTION

Obesity is a global public health problem. In the UK the prevalence of obesity has more than doubled in the last 30 years and today more than one-quarter of adults are living with obesity.^1^^,^^2^ Obesity causes cardiovascular disease (CVD) and diabetes, and a holistic approach which treats the underlying cause will improve long-term outcomes for patients.

In 2019, the NHS Long Term Plan highlighted obesity as a key focus for disease prevention, committing to providing targeted support and access to weight-management services in primary care.^3^ The UK government’s 2020 obesity strategy reinforces this call to action, placing primary care firmly at the centre of attempts to improve access for patients to effective weight-management services.^4^

Guidelines recommend that clinicians identify patients who are at high cardiovascular or metabolic risk, and promote weight loss to those with overweight or obesity giving relevant information on the associated health risks and setting realistic targets.^5^^,^^6^ This may increase patient engagement,^7^ but many clinicians cite insufficient confidence, knowledge, and skills to give specific recommendations.^8^ They also feel uncertain as to whether behavioural support will lead to weight loss with clinically significant improvements in disease risk.^9^^,^^10^

However, there is now good evidence that behavioural weight-management programmes suitable for use at scale in routine health care do lead to weight loss.^11^^–^^15^ While population-level benefits of weight loss in reducing cardiometabolic risk are well established, estimates of the magnitude of benefits seen on an individual level vary. Previous studies have suggested that there are reductions of 0.5–1 mmHg in blood pressure (BP), 0.1% glycated haemoglobin (HbA1c), and 0.02 mmol/L in total cholesterol for every 1 kg of weight loss.^16^^–^^20^ Conclusions, however, have often been limited by short duration of follow up and small sample sizes, with changes observed at only one follow-up timepoint; often the point of maximal weight loss.

This study aims to examine the association between weight loss and changes in cardiometabolic risk profile (BP, glycaemia, and lipids), at any time after referral to a weight-loss intervention, using data from an international cohort of participants in two large weight-loss trials with body weight measured over 24 months. This analysis takes into account the heterogeneity of individual weight loss trajectories. It aims to provide a realistic assessment of the changes in markers of cardiometabolic risk which may be expected as a result of intentional weight loss achieved through typical weight-loss interventions available in routine practice.

**Table:** How this fits in

Guidelines recommend that clinicians identify patients who are at high risk of cardiometabolic disease (for example, people with high blood pressure [BP]) and support weight loss in those with overweight or obesity. However, information quantifying the benefits of weight change for individuals, to guide these discussions in primary care, is lacking. This study demonstrates that weight loss achieved through referral to community weight-loss programmes, which are commonly accessible in primary care in the UK, can lead to clinically relevant reductions in BP and glucose regulation, especially in those at highest risk. An average weight loss of 5 kg could be expected to reduce glycated haemoglobin (HbA1c) by 3 mmol/mol in a person with type 2 diabetes, and reduce systolic BP by 2 mmHg in a person with hypertension. Larger weight losses give greater benefits, and reductions of around 10 kg are likely to be needed if a patient wishes to defer or reduce medication.

## METHOD

### Study characteristics

Data was pooled from two trials that tested the effectiveness of GP referral to community weight-loss programmes, in an observational analysis including all trial arms. Both trials and protocols have been published in full.^12^^,^^21^ The first,^21^ a parallel group, non-blinded, multicentre, randomised controlled trial recruited 772 adults with overweight or obesity from 115 primary care practices across Australia, Germany, and the UK. Participants were randomly assigned to either 12 months of standard care, or 12 months’ free membership to a weekly weight-management programme (WW, previously known as Weight Watchers), and were followed up with assessments at baseline, 2, 4, 6, 9, and 12 months. The second parallel group, non-blinded, multicentre, randomised trial,^12^ known as the WRAP trial, recruited 1269 adults with overweight or obesity from 23 primary care practices in England. Participants were randomly assigned to brief advice and self-help materials, a weight-management programme (WW) for 12 weeks, or the same weight-management programme for 52 weeks, and were followed up with assessments at baseline, 3, 12, and 24 months.

It has been difficult to draw conclusions about changes in cardiometabolic risk factors relevant to individual patients in UK primary care from previous systematic review evidence, because of the heterogeneity and intensity of included interventions and the lack of individual patient data with repeated measures over time.^17^^,^^19^^,^^22^ These two studies were selected to address these limitations, being as representative as possible of patients referred in routine primary care settings, with use of community weight-loss programmes that are currently widely available in UK practice and comparable pragmatic trial designs justifying pooling data from both studies.

Access to individual patient data, with multiple follow-up measurements at different timepoints, enabled this study to account for the variability of individuals’ weight-loss trajectories.

### Statistical analyses

Analyses were performed using Stata (version 16). The primary analyses assessed the association between the extent of weight change and changes in CVD risk factors. Data from all participants in both studies were pooled, and all analyses adjusted for baseline values time, sex, age, ethnicity, trial, and treatment group. A linear mixed effects modelling approach was used, with observations nested within participant to account for repeated measurements over time from the same participant.

Separate linear mixed effects regression models were fitted for changes in systolic blood pressure (SBP), diastolic blood pressure (DBP), glucose, glycated haemoglobin (HbA1c), total cholesterol, high-density lipoprotein (HDL), low-density lipoprotein (LDL), and triglycerides. For each individual participant, repeated measurements were available at different timepoints (any of 2, 3, 4, 6, 9, 12, or 24 months) for each outcome. Random slope models with an unstructured covariance structure were fitted to the data, and time (visit in months) was included as both a fixed and random effect to account for individual variability in the outcome over time. For all analyses, calculated weight change from baseline was used as the weight-change value for each timepoint, and the coefficient of weight change (at any time) yielded the primary outcome for each model. Change was calculated as observation 2 minus observation 1 (for example, weight at follow up minus weight at baseline). Model outputs are reported to one significant figure, or to the nearest integer for clinical examples.

Pre-specified subgroup analyses were performed according to participants’ baseline hypertension status, diabetes status, and plasma lipids for the outcomes of BP, glycaemia, and lipid profile, respectively. Hypertension status was defined by:

- GP record coding as hypertensive;
- taking antihypertensive medication;
- baseline SBP >140 mmHg; or
- baseline DBP >90 mmHg.

Diabetes status was defined by:

- GP record coding as diabetic;
- taking diabetes medication; or
- baseline HbA1c in diabetic range (≥48 mmol/mol).

Owing to inconsistent coding for hyperlipidaemia between datasets, clinical thresholds for baseline values were used to define abnormal plasma lipids:

- total cholesterol >5 mmol/L;
- HDL <1 mmol/L;
- calculated LDL >3 mmol/L; or
- TG (Triglycerides) >1.7 mmol/L.

For each outcome a separate linear mixed effects model was fitted for each subgroup, as models including an interaction term for subgroup status did not converge.

## RESULTS

The final dataset comprised 2041 participants with a mean (SD) age of 50 (SD 13.5) years, a mean weight of 90.6 (SD 14.8 kg, and a mean BMI of 32.7 (SD 4.1) kg/m^2^ at baseline. Three-quarters of participants were female, with just under 10% from ethnic minority groups (Table 1). Mean (SD) weight change across all timepoints was −4.3 (SD 6.0) kg — 51.2 to 21.5 kg), (range being maximum to minimum), equating to 4.7% total body weight loss (Table 2). Nearly half of participants achieved 5% weight loss measured on at least one occasion, and just under one-quarter lost >10% (Table 2).

**Table 1.** Baseline characteristics

	**Study 1^21^**	**Study 2^12^**	**Total sample**
***N*, total participants**	772	1269	**2041**

**Observations, *n***			
Weight	4033	3952	7985
BP	3853	3939	7792
HbA1c	1265	1314	2579
Blood test, other (glucose, lipid profile – *n*)	2251	1329	3580

**Sex, *n***			
Male	104	408	512
Female	668	861	1529

**Ethnicity, *n***			
White	482	1138	1620
Asian	23	26	49
Black	4	22	26
Other	68	41	109

Data not available	195	42	237

Age (years)^a^	47 (12.7)	53 (13.7)	50 (13.5)

Baseline BMI (kg/m^2^)^a^	31.4 (2.6)	34.5 (5.1)	32.7 (4.1)

Baseline Weight (kg)^a^	86.7 (11.5)	96.1 (17)	90.6 (14.8)

Baseline HbA1c (mmol/mol)^a^	38.2 (5.3)	41.3 (10.2)	39.2 (7.4)

Baseline Glucose (mmol/L)^a^	5.0 (0.8)	5.7 (1.7)	5.2 (1.2)

Baseline SBP (mmHg)^a^	124 (16)	133 (17)	128 (17)

Baseline DBP (mmHg)^a^	79 (9)	80 (10)	79 (10)

Baseline Total cholesterol (mmol/L)^a^	5.3 (1)	5.3 (1.1)	5.3 (1)

Baseline HDL cholesterol (mmol/L)^a^	1.4 (0.4)	1.7 (0.6)	1.5 (0.5)

Baseline LDL cholesterol (mmol/L)^a^	3.2 (0.9)	3.0 (1.0)	3.1 (0.9)

Baseline Triglycerides (mmol/L)^a^	1.5 (0.9)	1.5 (0.8)	1.5 (0.9)

Diabetes at baseline, *n* (%)	51 (6.6)	190 (14.9)	241 (11.8)

Hypertension at baseline, *n* (%)	92 (11.9)	631 (49.7)	723 (35.4)

a mean (standard deviation). BMI = body mass index. BP = blood pressure. DBP = diastolic blood pressure. HbA1c = glycated haemoglobin. HDL = high-density lipoprotein. LDL = low-density lipoprotein. SBP = systolic blood pressure.

**Table 2.** Mean weight loss

	**Observations, *n***	**Mean weight change, kg (SD)**	**Mean % change from baseline, (SD)**	**Number of people achieving ≥5% weight loss, *n* (% with recorded weight)**	**Number of people achieving ≥10% weight loss, *n* (% with recorded weight)**
Across all data points	5944	−4.3 (6.0)	−4.7 (6.4)	973 (47.7)	444 (21.8)
At 12 months	1267	−5.6 (7.2)	−6.1 (7.6)	596 (47.0)	317 (25.0)
At 24 months	1197	−3.6 (7.7)	−3.8 (8.1)	419 (35)	213 (17.8)

SD = standard deviation.

There were statistically significant improvements in all outcome measures (SBP, DBP, HbA1c, glucose, total cholesterol, LDL cholesterol, HDL cholesterol, and triglycerides) with weight loss (Table 3).

**Table 3.** Association of clinical variables with weight change

**Model**	**Change in outcome per kg weight change (95% CI)**	***P*-value**
**SBP, mmHg**	0.4 (0.32 to 0.45)	<0.001^a^
**DBP, mmHg**	0.3 (0.29 to 0.37)	<0.001^a^
**Total Cholesterol, mmol/l**	0.02 (0.01 to 0.02)	<0.001^a^
**HDL Cholesterol, mmol/l**	−0.003 (−0.006 to 0.0006)	0.015^a^
**LDL Cholesterol, mmol/l**	0.01 (0.01 to 0.02)	<0.001^a^
**Triglycerides, mmol/l**	0.02 (0.02 to 0.03)	<0.001^a^
**HbA1c, mmol/mol**	0.2 (0.13 to 0.22)	<0.001^a^
**Glucose, mmol/l**	0.02 (0.01 to 0.03)	<0.001^a^

a Statistically significant change at P<0.05. DBP = diastolic blood pressure. HbA1c = glycated haemoglobin.

HDL = high-density lipoprotein. LDL = low-density lipoprotein. SBP = systolic blood pressure.

On average across the whole population, each 1 kg of weight loss was associated with a 0.4 mmHg reduction in SBP and 0.3 mmHg reduction in DBP. For people with hypertension, greater reductions of 0.5 mmHg and 0.4 mmHg per kilogram were seen in SBP and DBP respectively, compared with 0.3 mmHg and 0.3 mmHg for people without hypertension (Table 4).

**Table 4.** Association of clinical variables with weight change: subgroup analysis according to baseline diabetes, hypertension and lipid status

**Subgroup**	**Variable**	**Change in outcome per kg weight change (95% CI)**	***P*-value**
**Hypertension**	SBP, mmHg	0.5 (0.36 to 0.60)	<0.001^a^
	DBP, mmHg	0.4 (0.29 to 0.43)	<0.001^a^

**No hypertension**	SBP, mmHg	0.3 (0.27 to 0.42)	<0.001^a^
	DBP, mmHg	0.3 (0.26 to 0.36)	<0.001^a^

**Diabetes**	HbA1c, mmol/mol	0.6 (0.35 to 0.79)	<0.001^a^
	Glucose, mmol/l	0.07 (0.03 to 0.12)	0.002^a^

**No diabetes**	HbA1c, mmol/mol	0.1 (0.09 to 0.14)	<0.001^a^
	Glucose, mmol/l	0.02 (0.01 to 0.02)	<0.001^a^

**Abnormal plasma lipids**	Total cholesterol, mmol/l	0.02 (0.01 to 0.02)	<0.001^a^
	HDL, mmol/l	−0.006 (−0.01 to −0.0003)	0.037^a^
	LDL, mmol/l	0.01 (0.005 to 0.02)	0.002^a^
	Triglycerides, mmol/l	0.04 (0.03 to 0.05)	<0.001^a^

**Normal plasma lipids**	Total cholesterol, mmol/l	0.02 (0.01 to 0.03)	<0.001^a^
	HDL, mmol/l	−0.003 (−0.006 to −0.0003)	0.028^a^
	LDL, mmol/l	0.01 (0.002 to 0.02)	0.011^a^
	Triglycerides, mmol/l	0.02 (0.01 to 0.02)	<0.001^a^

a Statistically significant change at P<0.05. DBP = diastolic blood pressure. HbA1c = glycated haemoglobin.

HDL = high-density lipoprotein. LDL = low-density lipoprotein. SBP = systolic blood pressure.

Each kilogram (kg) of weight loss was associated with a 0.2 mol/mol reduction in HbA1c in people with diabetes, each kg of weight loss was associated with a 0.6 mmol/mol reduction in HbA1c compared with a 0.1 mmol/mol reduction in people without diabetes (Table 4). Calculated examples of the change in BP and HbA1c that a patient might expect with different magnitudes of weight loss are presented in Table 5.

**Table 5.** Examples of average predicted changes in blood pressure and glycaemic control with weight change

**Variable**	**Example: variable change for X kg weight change**	**Variable change for every 5 kg weight change**	**Variable change for every 10 kg weight change**
**SBP** (all patients)	2 mmHg (1.6 to 2.2 mmHg) SBP change for 5 kg weight change	2 mmHg (1.6 to 2.2 mmHg) SBP change for 5 kg weight change	4 mmHg (3.2 to 4.5 mmHg) SBP change for 10 kg weight change
**SBP** (hypertensive subgroup)	1 mmHg (0.8 to 1.2 mmHg) SBP change for every 2 kg weight change	2 mmHg (1.8 to 3.0 mmHg) SBP change for 5 kg weight change	5 mmHg (3.6 to 6.0 mmHg) SBP change for 10 kg weight change
**DBP** (all patients)	3 mmHg (2.9 to 3.7 mmHg) DBP change for 10 kg weight change	1.5 mmHg (1.4 to 1.8 mmHg) DBP change for 5 kg weight change	3 mmHg (2.9 to 3.7 mmHg) DBP change for 10 kg weight change
**DBP** (hypertensive subgroup)	2 mmHg (1.4 to 2.2 mmHg) DBP change for 5 kg weight change	2 mmHg (1.4 to 2.2 mmHg) DBP change for 5 kg weight change	4 mmHg (2.9 to 4.3 mmHg) DBP change for 10 kg weight change
**HbA1c** (all patients)	1 mmol/mol (0.7 to 1.1 mmol/mol) HbA1c change for 5 kg weight change	1 mmol/mol (0.7 to 1.1 mmol/mol) HbA1c change for 5 kg weight change	2 mmol/mol (1.3 to 2.2 mmol/mol) HbA1c change for 10 kg weight change
**HbA1c** (diabetic subgroup)	3 mmol/mol (1.8 to 4.0 mmol/mol) HbA1c change for 5 kg weight change	3 mmol/mol (1.8 to 4.0 mmol/mol) HbA1c change for 5 kg weight change	6 mmol/mol (3.5 to 8.0 mmol/mol) HbA1c change for 10 kg weight change
**Glucose** (all patients)	0.2 mmol (0.1 to 0.2 mmol) glucose change for 10 kg weight change	0.1 mmol (0.1 to 0.1 mmol) glucose change for 5 kg weight change	0.2 mmol (0.1 to 0.3 mmol glucose change for 10 kg weight change
**Glucose** (diabetic subgroup)	0.6 mmol (0.2 to 1.0 mmol) glucose change for 8 kg weight change	0.4 mmol (0.1 to 0.6 mmol) glucose change for 5 kg weight change	0.7 mmol (0.3 to 1.2 mmol) glucose change for 10 kg weight change

DBP = diastolic blood pressure. HbA1c = glycated haemoglobin. SBP = systolic blood pressure.

The effect on plasma lipids was small, with changes of −0.02, −0.01, and 0.003 mmol/L in total, LDL, and HDL cholesterol respectively, and −0.02 mmol/L in triglycerides for every 1 kg of weight lost. A slightly greater change in HDL (0.006 mmol/L) and triglycerides (−0.04 mmol/L) was seen in those with abnormal plasma lipids at baseline (Table 4).

## DISCUSSION

### Summary

In individuals with overweight or obesity, weight loss achieved through referral to community weight-loss programmes does lead to clinically relevant reductions in BP and glycaemic control, especially in those at highest cardiometabolic risk. These data indicate that a 5 kg weight loss in people with hypertension will lead to an average reduction in SBP of 2 mmHg, increasing to 5 mmHg with 10 kg weight loss (Table 5). For people with diabetes, 5 kg and 10 kg weight loss may improve HbA1c by 3 mmol/mol and 6 mmol/mol respectively.

### Strengths and limitations

Two large pragmatic trials of community weight-loss programmes which are typical of the behavioural interventions available for referral in UK primary care were analysed. The findings are directly relevant for clinicians responding to NICE guidance that suggests clinicians should set specific goals for weight loss with individual patients, with realistic expectations for changes in associated cardiometabolic risk factors.^5^^,^^6^ Previous studies of weight loss and cardiovascular risk outcomes have used single timepoints at maximal observed effect,^17^ with meta-analyses based only on participants that completed the studies.^22^ As such, they cannot give an unbiased estimate of the typical association. The present approach addresses this limitation by performing a meta-analysis of individual patient data using mixed effects regression modelling to allow incorporation of all data for individuals across available timepoints. Multiple repeated observations over time were used to account for individuals’ trajectories of weight loss and regain, and the associated change in cardiometabolic outcomes, to provide a more realistic appraisal of the effects of weight change on an individual patient basis. This enables estimation of the average size of effect an individual might expect per kg of weight loss, rather than average effect of an intervention.

However, there are limitations. First, participants were predominately female (74.9%) and White (89.8%) This limits the extent to which the findings may be generalised to other patient groups, especially for patients of different ethnicities, as this may modify the effect of weight loss and could not be adequately assessed in these analyses.^23^^,^^24^

Second, the relatively small proportion of participants with diabetes at baseline (12%), and fewer HbA1c measurements available than for other outcomes, may make the findings relating to glycaemia less reliable.

Third, it remains possible that a confounder correlated with weight loss, but not caused by it, explains the association reported between weight loss and improvements in cardiometabolic risk factors (although this seems unlikely). Changes in concomitant medication use in this model could not be accounted for (a frequent limitation among meta-analyses describing BP/HbA1c change with weight loss).^17^^,^^18^^,^^22^ In pragmatic trials in routine practice such as these, GPs may have added or removed medications in response to changes in BP or glycaemia, thus underestimating the strength of the association, and there are no data available to assess whether this is the case.

### Comparison with existing literature

Previous reviews have reported up to a 1 kg:1 mmHg relationship between change in weight and change in BP,^17^^,^^18^^,^^22^ approximately double that reported here. One such meta-analysis of randomised controlled trials reporting a 1 kg:1 mmHg ratio assessed outcome measures at the timepoint of maximal observed effect (a mean duration of 35.3 weeks), which may represent acute but not more sustained changes. The present study, however, uses repeated measures for individuals up to 24 months from commencing weight loss.^17^ Additionally, as in this study’s findings, the effect of weight loss on BP was larger in patients with pre-existing hypertension. People with hypertension formed a larger proportion of the study populations in the meta-analysis (half the included patients, compared with 35% in the present study), which may contribute to the larger effect found. The demographics of the population studied (mean age 50 years; majority female participants) is more representative of the people currently being referred to community weight-loss programmes in the UK (median age 49 years; 90% female)^25^^,^^26^ than people commonly recruited to trials such as those included in Neter and others’ meta-analysis (which studied a younger, predominately male [64%] population).^17^

This study’s findings suggesting that a 10 kg weight loss is associated with a 6 mmol/mol (0.5%) improvement in HbA1c in patients with type 2 diabetes align with data presented from observational analyses of the Look AHEAD study of individuals with type 2 diabetes and obesity over 1 year.^27^ The greater benefit demonstrated in fasting glucose in the short term in Look AHEAD may have been observed owing to the worse glycaemic control of their study population at baseline. In Look AHEAD, mean (SD) baseline HbA1c was 7.28 (SD 1.17)%, and mean baseline fasting glucose was 8.5 (SD 2.5) mmol/L. This is compared with the subgroup of participants with type 2 diabetes in the present study, where mean (SD) baseline HbA1c was 6.9 (SD 1.35)%, and mean baseline fasting glucose was 7.4 (SD 2.6) mmol/L.

A previous meta-analysis reported a 0.2 mmol/L reduction in total cholesterol per 10 kg weight loss, which is consistent with this study’s finding.^19^ This 3–5% reduction in total cholesterol observed — while statistically significant — is less than one-quarter of the expected reduction from starting treatment with a statin.^28^ While this alone is unlikely to significantly reduce cardiovascular risk for an individual, a higher BMI is associated with higher CVD risk independent of cholesterol status; as such, any weight reduction is likely to be of benefit.^29^^,^^30^ In addition, population-level benefits remain; it is estimated that a reduction of only 2% in the total cholesterol level of each person in the population could lead to 42 fewer deaths per 100 000 people from coronary heart disease over 10 years.^31^^,^^32^

### Implications for research and practice

The need to support weight loss in people with overweight or obesity and at high cardiometabolic risk is now widely recognised, and is a core recommendation in national guidance and the NHS Long Term Plan.^3^^,^^5^ Despite this, pharmacotherapy remains the predominant management strategy for treatment and prevention of cardiometabolic disease: in the recent Health Survey for England >90% of people with diabetes or CVD reported being on ≥1 prescribed medications,^33^ while only a quarter of people with overweight or obesity reported having ever received a health professional’s advice to lose weight.^34^

This study’s findings show that modest weight loss is associated with improved cardiometabolic risk factors, and that more weight loss is associated with greater changes. How much weight, then, should patients aim to lose? A threshold of 5% body weight loss is often quoted as a target from which clinically significant results may be expected.^6^ This is close to the average weight loss seen in this study (4.3 kg; 4.7% mean percentage body weight change) and was associated with some small but clinically relevant benefits; for example, a 2.5 mmol/mol (95% confidence intervals [CI] = 1.5 to 3.4 mmol/mol) reduction in HbA1c in patients with diabetes. With >40% of people referred to a 12-week community weight-loss programme maintaining 5% weight loss at 1 year, this presents an appropriate and reasonable initial goal.^12^ Greater weight loss, however, brings bigger benefits.

For an individual patient hoping to avoid starting or escalating medication usage in response to a diagnosis of raised BP or impaired glycaemia, weight loss of ≥10% (equating to 9–10 kg weight loss for the average patient in this study population; approximately one standard deviation above the average weight loss seen) may be required to give a realistic possibility that it will reduce their need for medication. Metformin monotherapy can be expected to reduce HbA1c by around 12 mmol/mol,^35^ while starting an antihypertensive agent reduces average SBP by 9.1 mmHg, and DBP by 5.5 mmHg.^36^

In the studies analysed here, 26% of participants referred to community weight-loss services lost ≥10% of their body weight (≥9–10 kg weight loss). These results suggest that weight loss of this magnitude could confer benefits in BP and HbA1c equivalent to approximately half that of commencing drug monotherapy with antihypertensive agents or metformin. This is not an insignificant amount of weight for a patient to lose, and is far greater than the average weight loss of 1 kg observed when patients are given only brief advice to lose weight by their GP without referral for additional support.^11^ This reinforces the message that clinicians should focus on directing patients towards effective support if they are aiming to achieve these greater weight losses. It is difficult to predict which patients will have most success from weight-management programmes, except based on early weight-loss achievements once attempting the programme.^37^ Around one in seven people following this kind of intervention will achieve ≥10% body weight reduction at 1 year in the 12-week programmes that are commonly commissioned.^11^^,^^12^ However, this increases to one in three people referred to a 52-week programme, suggesting that repeat (or extended) referrals for people who are achieving weight loss, which have been shown to be cost effective,^12^ are clinically beneficial.

This study has focused on cardiometabolic risk, but the individual and population benefits of this achievable weight loss are likely to be much wider, from improvements in comorbidities (such as reduction of pain and improvements in mobility in osteoarthritis)^38^ to reductions in primary care resource utilisation.^39^

A recent study demonstrated that excess weight accounts for an estimated 11% (£229 million) of all primary care consultation costs and 20% (£384 million) of prescription medication costs, with each BMI increase of 2 kg/m^2^ (in females with BMI >20 kg/m^2^) associated with 5.2% (CI = 4.8–5.6) and 9.9% (CI = 9.2–10.6) higher mean annual consultation and prescription medication cost respectively.^39^ Helping patients to reduce their weight could not only improve their health but also reduce pressures on primary care. But to realise this potential, and that of population level prevention of CVD, there is a need to actively offer referrals to weight-loss programmes and engage far more widely than current figures suggest is being done. The type of interventions in the trials in this study are widely available for GP referral and use in the NHS.

In summary, moderate weight loss of 5–10 kg can achieve clinically meaningful reductions in markers of cardiometabolic risk and may offer the opportunity for patients to defer or reduce medication. These data provide clinicians with the information they need to discuss this option in their consultations.
